# Systemic Mesenchymal Stem Cell-Derived Exosomes Reduce Myocardial Infarct Size: Characterization With MRI in a Porcine Model

**DOI:** 10.3389/fcvm.2020.601990

**Published:** 2020-11-16

**Authors:** Christopher J. Charles, Renee R. Li, Teresa Yeung, Stephane M. Ibraham Mazlan, Ruenn Chai Lai, Dominique P. V. de Kleijn, Sai Kiang Lim, A. Mark Richards

**Affiliations:** ^1^Cardiovascular Research Institute (CVRI), National University Heart Centre, Singapore, Singapore; ^2^Department of Surgery, Yong Loo Lin School of Medicine, National University of Singapore, Singapore, Singapore; ^3^Department of Medicine, Christchurch Heart Institute, University of Otago, Christchurch, New Zealand; ^4^Institute of Medical Biology, Agency for Science, Technology and Research (A^*^STAR), Singapore, Singapore; ^5^Department of Vascular Surgery, University Medical Centre, Utrecht, and Netherlands Heart Institute, Utrecht, Netherlands

**Keywords:** mesenchymal stem cells, exosomes, porcine (pig) model, myocardial infarction, left ventricular remodeling, cardiac fibrosis, cMRI

## Abstract

The observations that mesenchymal stem cells (MSCs) exert cardiac protection and repair via their secretome with the active component(s) identified as exosomes underpinned our test of the efficacy of MSC exosomes in a porcine model of myocardial infarction (MI) when administered systemically by the convenient method of intravenous (IV) bolus injection. Results show that 7 days of IV exosomes results in clear reduction (30–40%) of infarct size measured at both 7 and 28 days post-MI, despite near identical release of hs Troponin T. Together with reduced infarct size, exosome treatment reduced transmurality and lessened wall thinning in the infarct zone. Exosome treated pigs showed relative preservation of LV function with significant amelioration of falls in fractional wall thickening compared with control. However, global measures of LV function were less protected by exosome treatment. It is possible that greater preservation of global LV function may have been attenuated by increased cardiac fibrosis, as T1 values showed significant increase in the exosome pigs compared to control particularly in the infarct related segments. Taken together, these results show clear effects of IV exosomes administered over 7 days to reduce infarct size with relatively preserved cardiac function compared to control treated infarct pigs.

## Introduction

The potential of cell-based therapies to stimulate cardiac regeneration following myocardial infarction (MI) has been investigated for two decades with many pre-clinical studies showing promise but mixed results from clinical studies to date and some controversy resulting from fraudulent publications (1). Stem cell types such as mesenchymal stem cells (MSC) derived from bone marrow have been shown to have potent effects through multiple mechanisms (2) despite a number of studies demonstrating poor engraftment with minimal evidence of differentiation of injected cells in the infarct zone (3–5). Furthermore, engrafted cells only survive for days whereas beneficial effects can be observed over weeks (5). These observations led to the hypothesis that MSCs exert their therapeutic effects via the paracrine effects of multiple secreted cytokines and growth factors that reduce myocardial injury and promote repair (6).

Subsequent studies have demonstrated that conditioned medium from cultured MSCs over-expressing Akt gene injected into myocardium reduced infarct size in a rodent model of MI to the same degree as injection of the cells themselves (7). We have demonstrated in a pig model of ischemia/reperfusion (I/R) injury that MSC conditioned medium administered by either intravenous (IV) or intracoronary routes reduced infarct size measured 4 h post-I/R and also improved both systolic and diastolic function (8). In a follow-up study, IV MSC conditioned medium administered for seven days improved myocardial function measured at three weeks after MI induced by permanent coronary artery ligation in pigs (9). Further, our group was the first to show that exosomes carried the active component(s) of MSC conditioned medium and that infused MSC exosomes reduce I/R injury in mice (10, 11). Together, these and other studies provide key evidence for the paracrine hypothesis whereby MSC therapeutic efficacy is mediated primarily via the MSC secretome. As reviewed by our group (12), MSC exosomes carry many of the therapeutic agent(s) in MSC secretion.

MSC exosome preparations are now widely shown to be heterogenous mixtures of different extracellular vesicle (EV) types of similar size ranges and consist of at least three types of EV types including exosomes (12). Currently, it is not technically possible to isolate specific EV types and therefore the International Society for Extracellular Vesicles in their position paper, MISEV2018, recommends that the term EV be used for EV preparations of unknown specificity and that different descriptors such as “small” could be used to stratify different EV preparations (13). ISEV, ISCT, ISBT, and SOCRATES have defined preparations of 50–200 nm EVs secreted by MSCs as MSC-small EV (MSC-sEV) preparations (14). Therefore, MSC exosomes are essentially “MSC-sEV preparations”

Use of exosomes as the therapeutic modality offers potential for upscaling and standardizing production of stable exosomes that may be able to be used as “off the shelf” therapy. Further studies addressing route, dose, and duration of delivery of exosomes in relevant pre-clinical models are necessary. Accordingly, we have determined the cardioprotective effects of IV MSC-derived exosomes administered over 7 days post-MI in a pig model of permanent coronary artery ligation.

## Materials and Methods

The study protocol was approved by the Institutional Animal Care and Use Committees of National University of Singapore. Twenty pigs female Yorkshire x Landrace pigs (41–62.2 kg) were housed individually receiving standard care.

### Preparation of Exosomes

MSC-sEVs were prepared from culture medium conditioned by a clonal immortalized E1-MYC 16.3 human embryonic stem cell-derived MSC line as previously described (15). The cells were first grown in DMEM with 10% fetal calf serum (16). When the culture was 80% confluent, the cells were washed with PBS and grown in a chemically defined medium for 72 h. The defined medium was prepared as follow: 480 mL DMEM (31053, Thermo Fisher), 5 mL NEAA (11140-050, Thermo Fisher), 5 mL L Glutamine (25030-081, Thermo Fisher), 5 mL Sodium Pyruvate (11360, Thermo Fisher), 5 mL ITS-X (51500-056, Thermo Fisher), 0.5 mL 2-ME (21985-02, Thermo Fisher). This was supplemented with 0.1 mL bFGF (0.5 ng/μl 0.2%BSA in PBS(+) and 0.005 mL PDGF) [100 ng/μl PBS(+)]. These components were obtained as follows: Bovine Serum Albumin or BSA (A9647, Sigma-Aldrich), PDGF (100-00 AB CYTOLAB), bFGF (13256-029, Thermo Fisher) and PBS(+) (14040-133, Thermo Fisher). The culture medium was enriched for exosomes by tangential flow filtration using a membrane with a molecular weight cut-off of 100 kDa (Sartorius, Gottingen, Germany) before sterile filtration with a 0.22 μm filter (Merck Millipore, Billerica, MA, USA). The preparation was assayed for protein concentration using a NanoOrange Protein Quantification Kit (Thermofisher Scientific, Waltham, MA, USA). Purified exosomes were stored frozen (-80°C) as aliquots equating to 1000 ug protein prior to administration to pigs as below.

### Characterization of Exosomes

**Figure 1:**
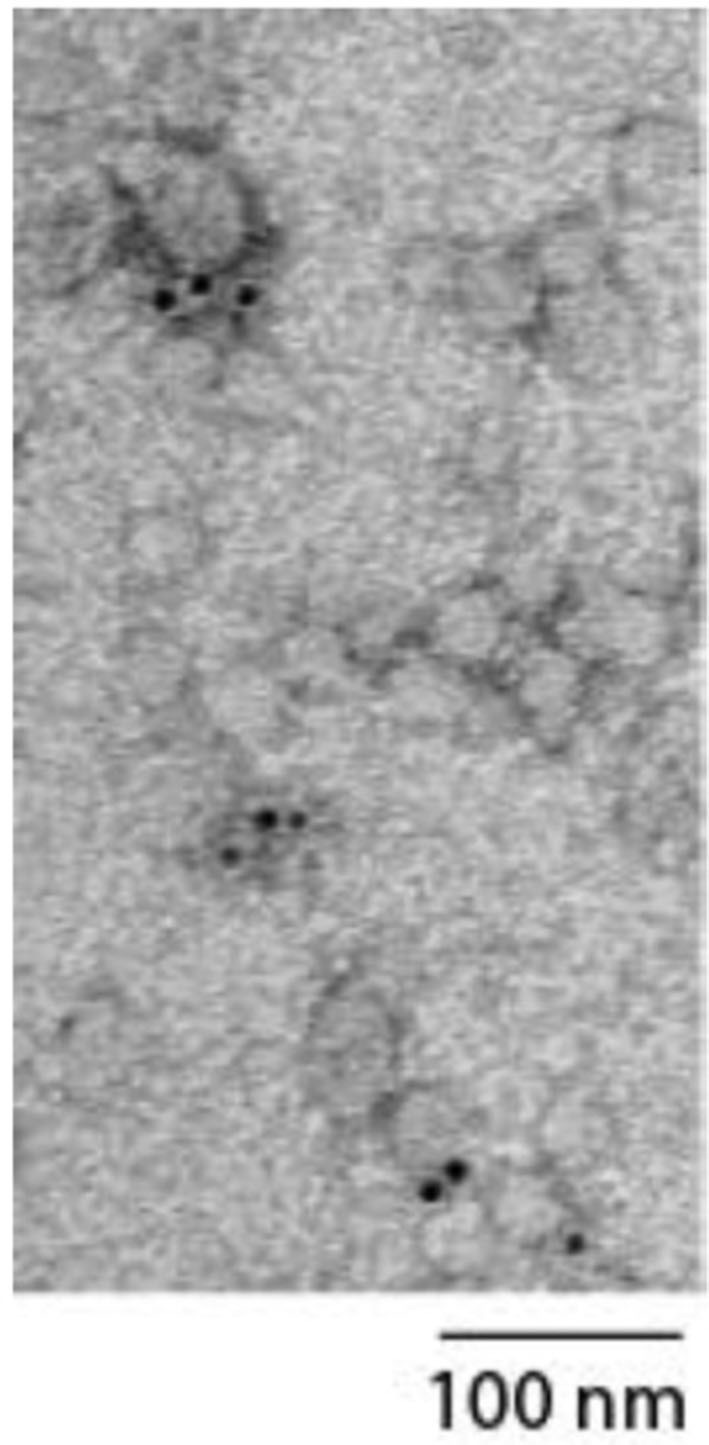
Electron micrograph showing particles measuring between 50 and 100 nm labeled with gold beads indicating the presence of CD81+ particles.

MSC-sEV preparations prepared using the above protocol have been extensively characterized for size, density, exosome-associated markers, proteins, RNA and enzyme activities (10, 17–19). For this study, the preparation was analyzed in accordance with the MSC-sEV identity metrics recommended by expert members of the ISEV, ISCY, ISBT, and SOCRATES (14). The MSC-sEV preparation was determined to have particles with a modal size of 137.6 nm by Nanoparticle Tracking Analysis on a ZetaView instrument (Particle Matrix GmbH, Germany) using the parameters (sensitivity = 90, shutter = 70, frame rate = 30, min brightness = 25, min area = 5, max area = 1000). The MSC-sEV preparation was determined to have 5.72 ng cholesterol/ug protein using a commercially available assay kit, Amplex Red Cholesterol Assay kit according to the manufacturer's instructions (A12216 Thermofisher Scientific). The enzymatic activity of a common MSC surface antigen, CD73 in the preparation was determined to be 34.94 mU/ug protein. Briefly, the assay was performed by first replacing the phosphate buffer in MSC-sEV preparation with 20 mM Tris buffer, pH 7.4. Briefly, 5 μg MSC-sEV sample was pipeted into a pre-wetted 96-well membrane filter plate with MWCO of 100 kDa (Pall, #8036) and the buffer was then removed by vacuum filtration. The sample was washed 3 times using 250 μL of 20 mM Tris buffer per well each time. After removal of the last wash, CD73 enzymatic assay was initiated by adding 100 μL of 800 μM AMP in 20 mM Tris buffer, pH 7.4 to each well. The sample was incubated at 37°C, 300 rpm for 60 min on a microplate mixer. After incubation, the amount of phosphate formed by CD73-mediated hydrolysis of AMP was determined using a PiColorLock^TM^ Gold Phosphate Detection System (Innova Biosciences, #303-0030). A phosphate standard series of 0-25 μM was prepared using a stock phosphate solution provided in the kit. 60 μL from MSC-sEV sample or the phosphate standard solutions were each transferred to a well in a transparent 96 well plate that was pre-loaded with 15 μL of the “Gold Mix” in each well. The “Gold Mix” was prepared using the stock reagent provided in the PiColorLock^TM^ Gold Phosphate Detection System. The plate was gently shaken to mix the contents in each well and incubated for 15 min at 25°C. The absorbance signal at 635 nm was measured in a microplate reader. All the assays were performed in triplicates. To visualize if CD81, a tetraspanin membrane protein was present on particles in the preparation, immunoelectron microscopy was performed. Glow-discharged EM grids coated with formvar-carbon (EMS) were floated on a 20 ul drop of MSC-sEV preparation. Excess fluid was carefully removed with filter paper. The grid was then incubated with mouse monoclonal anti-CD81 antibody (Santa Cruz Sc-7637) followed by Goat anti-mouse secondary antibody coupled with 6 nm gold (EMS) before fixing with 1% glutaraldehyde (EMS). The grid was washed and embedded in a thin film of Uranyl Acetate-methylcellulose (mixture of 4% Uranyl acetate and 2% methycellulose in 1:9 ratio). The sample was analyzed by JEOL transmission electron microscope (JEM-1010) operated at 80 kV and equipped with SIA model 12C 4K CCD camera. Particles of between 50 and 100 nm with gold beads were observed indicating the presence of CD81+ particles as shown in Figure 1. The size of the particles was less than the modal size determined Nanoparticle Tracking Assay because of dehydration during sample preparation.

### MI Study Protocol

All 20 pigs underwent baseline (day 0) cardiac magnetic resonance imaging (CMRI) under general anesthesia prior to sternotomy surgery. Pigs underwent repeat anesthesia for CMRI on Days 7 and 28 post-MI. For CMRI, pigs were pre-medicated with intramuscular (IM) ketamine (10 mg/kg), atropine (0.04 mg/kg) and midazolam (0.6 mg/kg), intubated, ventilated, and maintained with inhalation isoflurane. During MRI, immediately prior to contrast administration, pigs received IM diphenhydramine (1 mg/kg). Following day 0 imaging, pigs were switched to total IV anesthesia (midazolam 2.5-4 mg/kg/h, alfentanil 250-400 μg/kg/h and pancuronium 0.25-0.3 mg/kg/h) for surgical sternotomy and left circumflex (LCX) coronary artery ligation procedure. The LCX was carefully dissected in the region approximately midway across the base of the heart directly under the left atrial appendage and the artery ligated prior to any major branches of the LCX. Other medications included oral amiodarone (400 mg/day loading dose day−10 to day−1 then 200 mg/day days 1-28), oral clopidogrel (600 mg loading dose day−1 followed by 75 mg daily from day 1–28), augmentin (15 mg/kg IV then 625 mg orally for 5 days) and fentanyl transdermal patches (2 μg/kg/h for 4 days). Following LCX ligation ten pigs were randomized to receive 1000 ug protein equivalent of exosomes administered after dilution to 50 ml with 0.9% saline as an IV bolus 60 min after ligation followed by twice daily bolus exosome treatment IV injections for seven days. The remaining group of ten pigs served as a control group receiving IV bolus of saline following MI. Bolus injections and blood samples (below) were drawn from an indwelling jugular vein catheter placed via a neck cut-down soon after ligation with a connecting tube tunneled to the dorsal base of the neck.

CMRI was performed using a Siemens 3T MRI scanner (MAGNETOM Skyra). Our standard CMRI protocol included cine imaging [global and regional left ventricular (LV) systolic function including LV volumes, mass, and LV ejection fraction (LVEF)], Early Gadolinium Enhancement (EGE) and Late Gadolinium Enhancement (LGE) imaging after the injection of Magnevist at a dose of 0.2 mmol/kg, and T1-mapping from Modified Look-Locker Imaging (MOLLI). MRI analysis was performed using Segment v2.2 R6338 (http://segment.heiberg.se). Epi- and endocardium contours were segmented from cine images. Segmented contours were overlaid onto LGE images and infarcts were delineated within the myocardium using Otsu segmentation (20). T1 values were used as an index of cardiac fibrosis following segmentation of the LV based on the American Heart Association 17 segment model. For determination of end systolic (ES) LV wall thickness (WT_ES_) a mid-slice MRI cine was divided into 12 sectors with sectors 4 and 5 being centered on the infarct region. Fractional wall thickening was determined on the same 12 sectors of the mid-slice and determined as (WT_ES_-WT_ED_)/WT_ED_, with WT_ED_ being wall thickness at end diastolic.

Ethylenediaminetetraacetic acid blood samples drawn at baseline and then daily for the first seven days post-MI were centrifuged and plasma stored at−80°C prior to assay high sensitivity (hs) Troponin T using the Roche Elecsys platform. Following the day 28 MRI scan, hearts were harvested immediately post-mortem (PM), LV trimmed of all other tissues, and ~4–5 equal thickness LV cross-sectional rings cut from base to the apex of the heart, photographed top and bottom to allow subsequent sizing of the infarct region as a percentage of the LV by planimetry. Given that infarcts were 28 days old they were easily distinguished from viable myocardium based on color difference without need for further processing. Samples of LV tissue in the infarct region were harvested PM from four control and four exosomes treated pigs and fixed in buffered 10% formaldehyde prior to paraffin embedding. Haemotoxylin and Eosin (H&E) and picrosirius red stains were performed by standard methods.

### Statistics

Pigs were randomized for control or exosomes treatment prior to baseline MRI and surgery for ligation induced MI. Results are expressed as mean ± standard error of mean (SEM). Two-way analysis of variance (ANOVA) with time as a repeated measure was used to determine time x treatment interaction differences between exosomes and control groups. Fisher's protected least significant difference (LSD) method was used to determine individual time-points significantly different from time-matched controls, including assessment of any potential baseline differences. *P* < 0.05 was the threshold for statistical significance.

## Results

**Figure 2:**
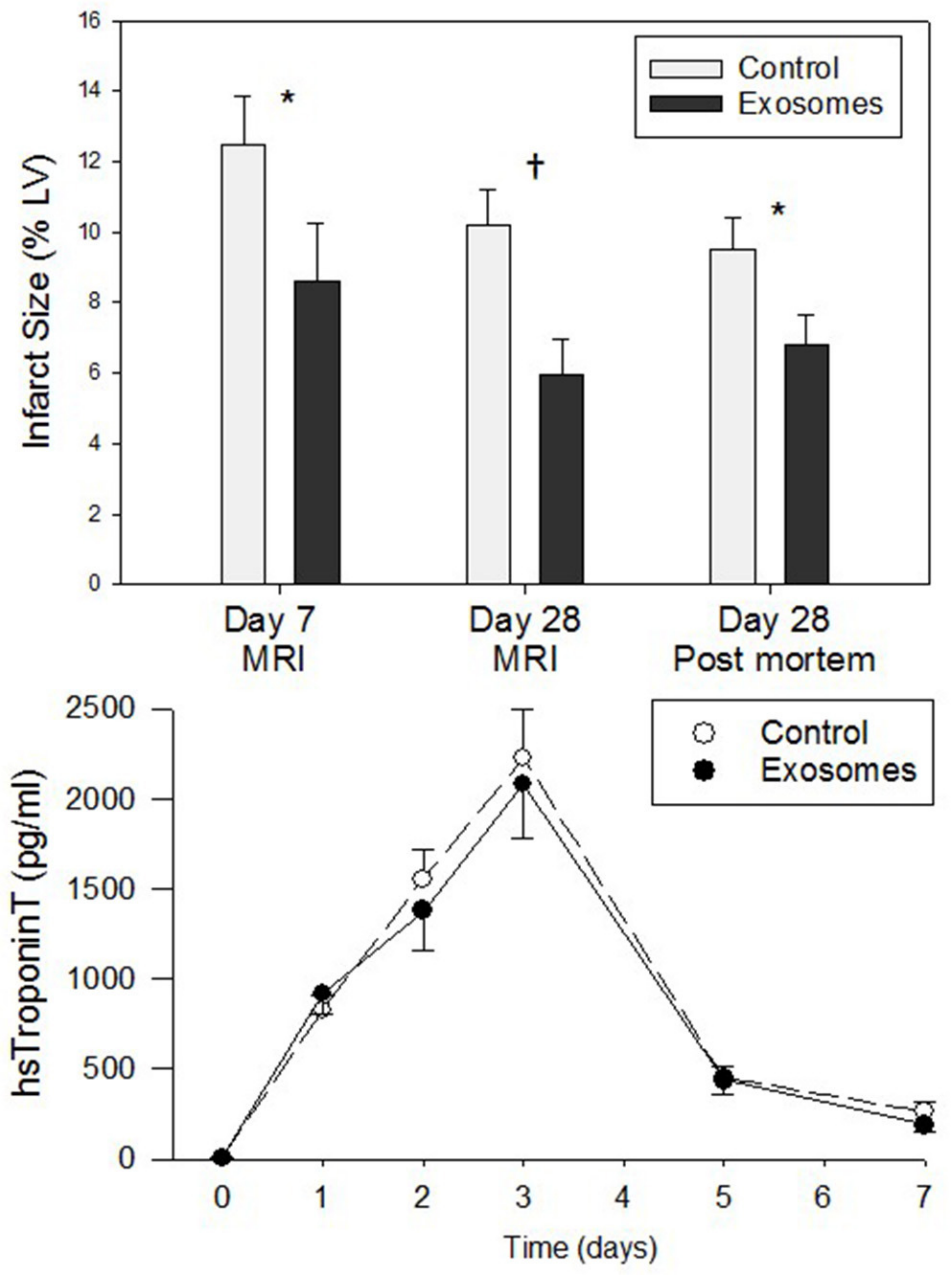
**(Top)** Infarct size measured by cardiac magnetic resonance imaging (MRI) or post mortem left ventricle (LV) ring planimetry in ten pigs receiving 7 days intravenous administration of exosomes following myocardial infarction (MI) by means of left circumflex coronary artery ligation and ten pigs vehicle control treated MI pigs. **(Lower)** High sensitivity (hs) TroponinT plasma levels for 7 days post-MI in exosome (•) vs. control (°) treated pigs. Values shown are mean ± SEM. Significant differences at time-matched points between the exosomes and control pigs are indicated as follows: *p < 0.05 and ^†^*p* < 0.01.

All ligations of the LCX resulted in permanent ischemia with MI as evidenced by pallor of the myocardium on the LV surface, ECG changes including ST segment elevation and increases in plasma hs Troponin T measured over the subsequent 7 days (Figure 2). Troponin T levels increased initially to peak three days post-MI and thereafter return toward baseline levels with identical time-course apparent for both control and exosomes treated pigs (no significant difference, Figure 2, lower panels). Despite virtually identical peak (day 3) level and area under curve for hs Troponin T response, resultant infarct size assessed by MRI was significantly reduced by exosome treatment when measured at both day 7 (7.61 ± 1.10% vs. 12.52 ± 1.38% for exosomes and control respectively, *p* < 0.05) and day 28 (5.96 ± 0.99% vs. 10.23 ± 1.02%, *p* < 0.01). The day 28 MRI results were confirmed by assessing infarct size by the heart ring PM method (6.82 ± 0.84% vs. 9.53 ± 0.88%, *p* < 0.05). This represented a reduction in infarct size of ~30–40% (Figure 2, top panel).

**Figure 3:**
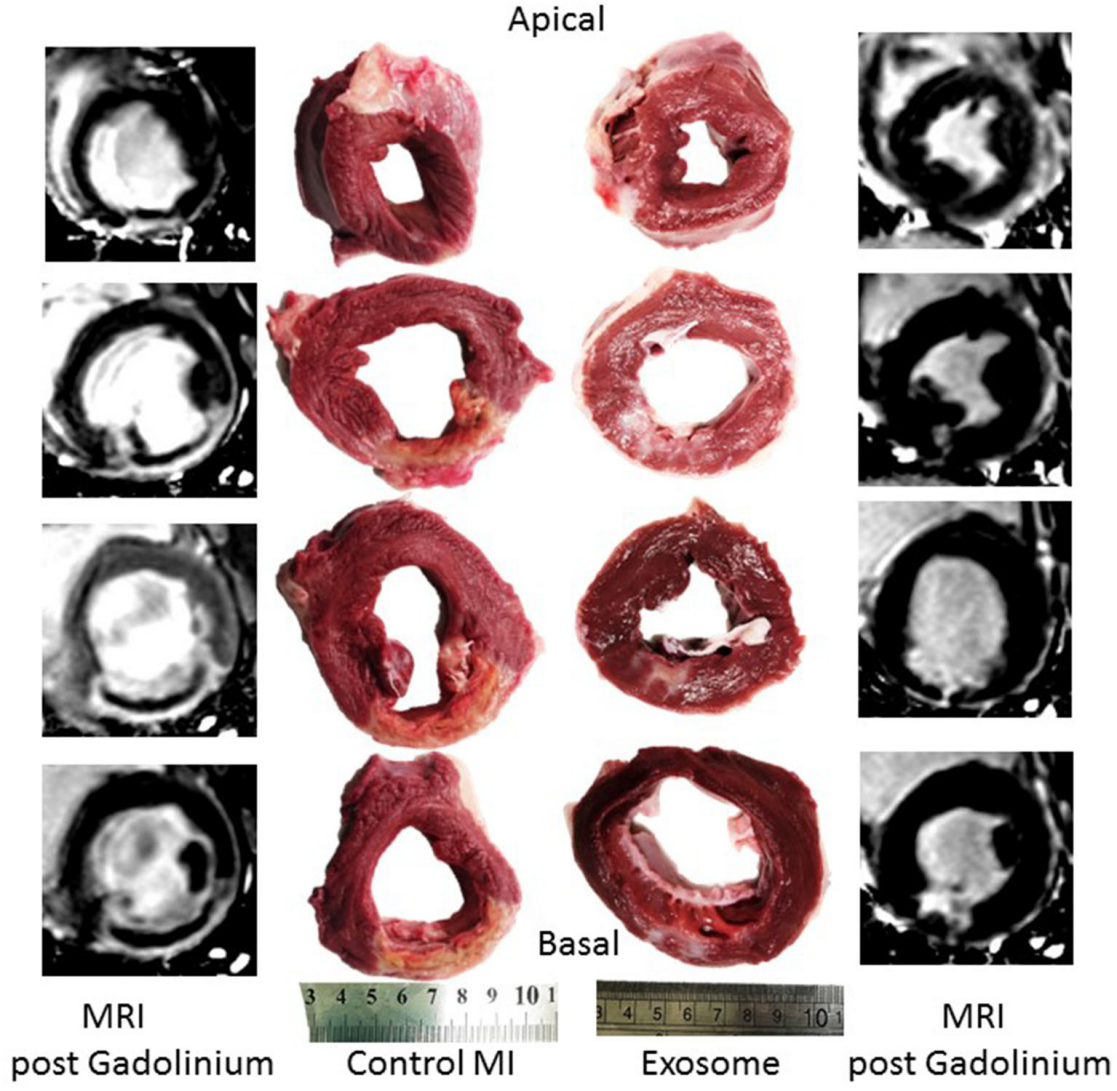
Examples of MRI post-gadolinium short axis images of left ventricle 28 days post-MI and corresponding gross pathology from post mortem left ventricular rings showing infarct vs. normal myocardium from a representative exosomes treated and control treated MI pig.

**Figure 4:**
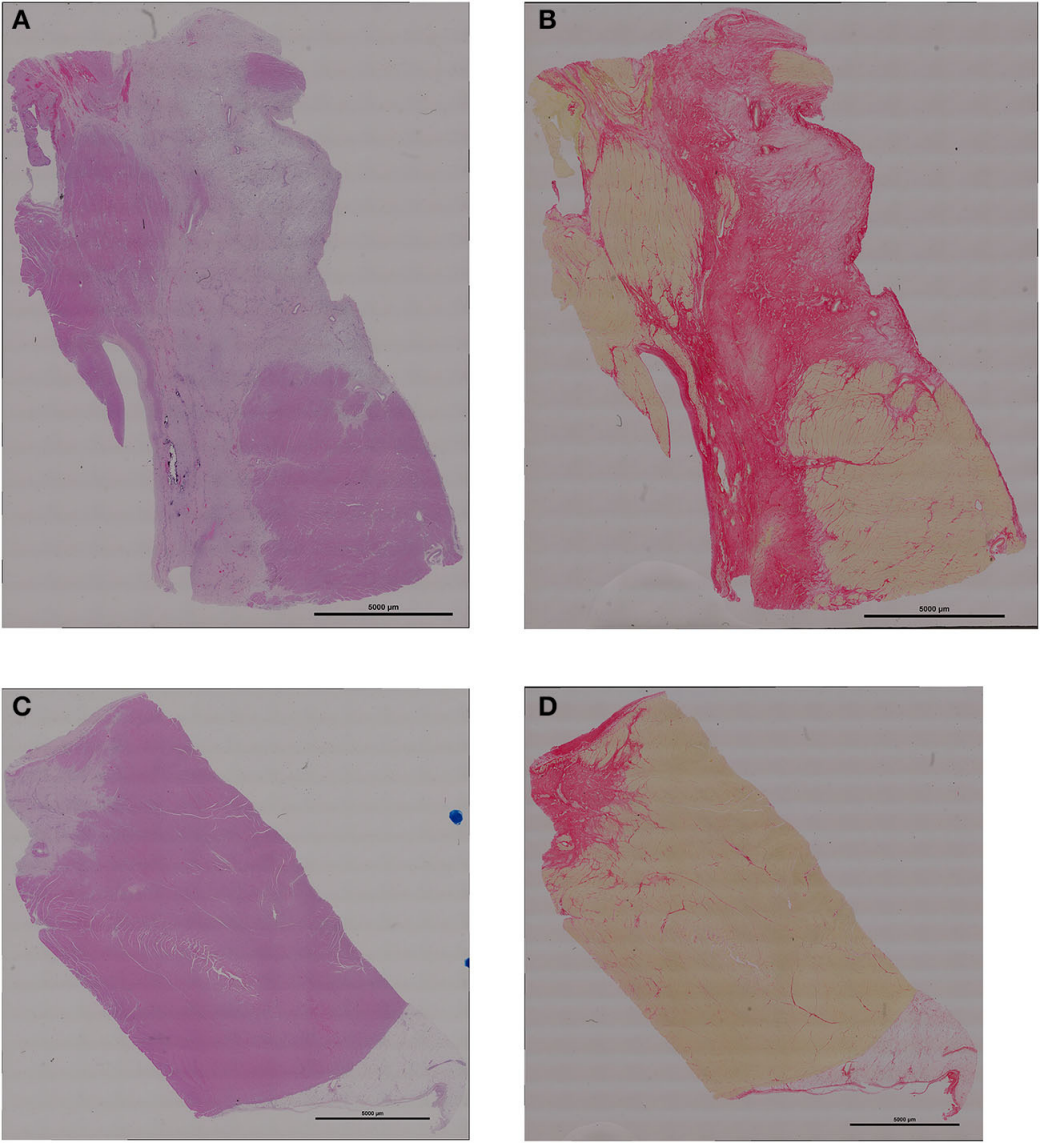
Representative examples of histology of the infarct region **(A)** H&E staining from control treated pig; **(B)** picrosirius red staining from control pig; **(C)** H&E from exosomes treated pig; **(D)** picrosirius red from exosomes pig. Scale bar for all images is 5000 um long.

Differences in infarct size between the exosome treated and untreated pigs were obvious on both MRI, day 7 (images not shown) and day 28 (Figure 3), and gross histological examination of the PM LV rings as shown in Figure 3 by exemplar MRI images and PM photographs of heart rings, LCX ligation in control pigs resulted in significant transmural infarcts involving ~25–35% of the circumference of the LV wall extending from the basal slice through to 3-4 of 5 LV rings with obvious wall thinning and well developed scar tissue. In contrast, exosome treated infarcts were much smaller and were generally not transmural, with little evidence of wall thinning. These findings were reinforced by histopathology with exemplar slides from a control and exosome infarct region stained for H&E and picrosirious red shown in Figure 4.

**Figure 5:**
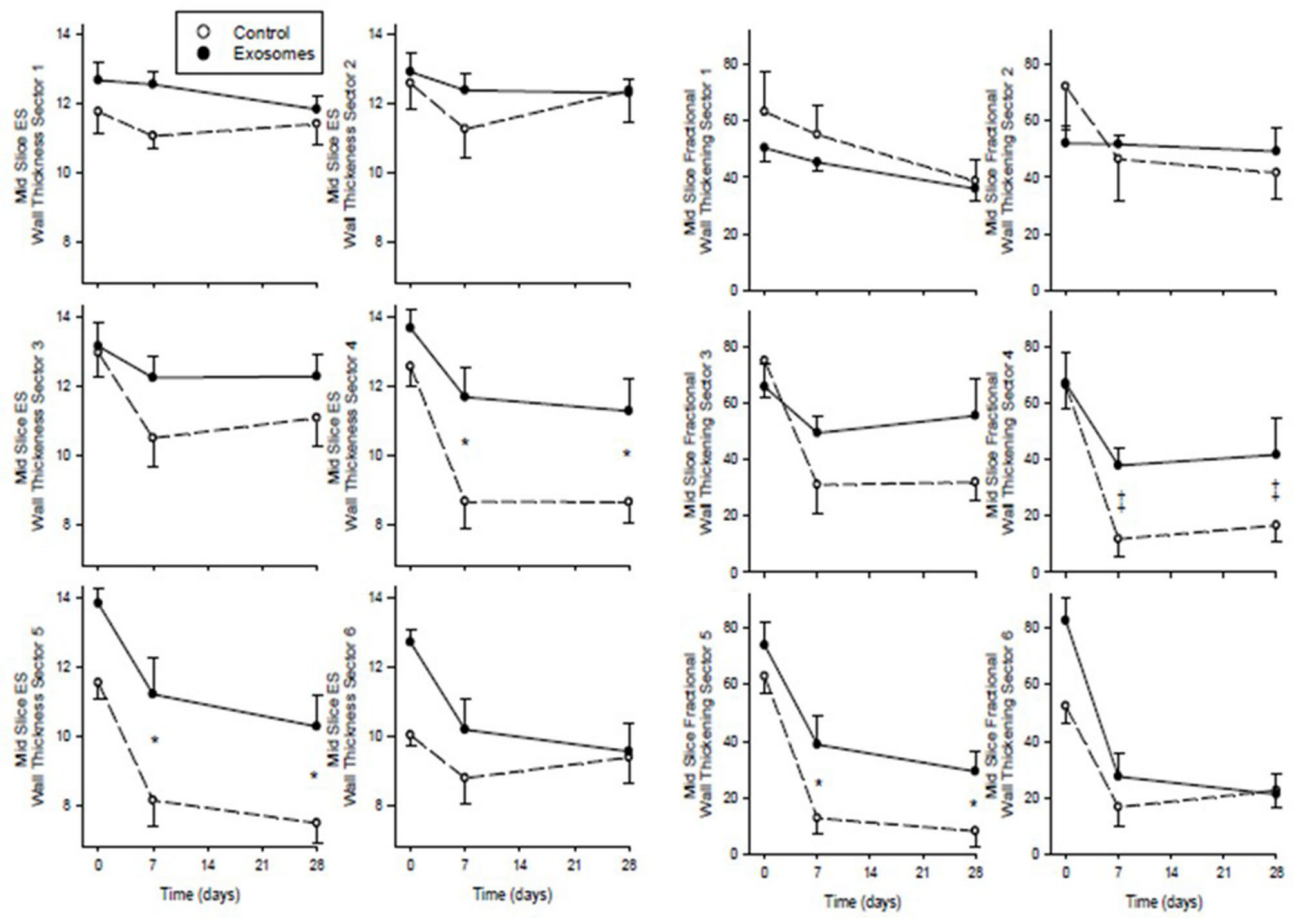
Serial mid-slice end systolic (ES) wall thickness (left) and fractional wall thickening (right) measured by MRI across 6 sectors of the left ventricle spanning the infarct in ten pigs receiving 7 days intravenous administration of exosomes (•) following MI and ten pigs vehicle control (°) treated pigs. Values shown are mean ± SEM. Significant differences at time-matched points between the exosomes and control pigs are indicated as follows: *p < 0.05 and ‡*p* < 0.001.

The significant wall thinning was confirmed by MRI measurement of wall thickness. Figure 5 (left panels) show mid-slice WT_ES_ measured across six of the 12 sectors measured. WT_ES_ in control MI pigs was significantly reduced from approximately 50% from baseline when measured 7 and 28 days post-MI in sectors 4 and 5, which are directly centered on the infarct zone, with significantly less wall thinning apparent in the exosome treated pigs at both sector 4 (*p* = 0.009 by ANOVA) and sector 5 (*p* = 0.004). The degree of preservation of wall thickness by exosome treatment was similar at both day 7 and day 28 post-MI. There was no difference between control and exosome treated pigs for WT_ES_ across the other sectors outside the infarct zone (sectors 1-3 and 6 shown in Figure 5) or across sectors 7-12 (data not shown). Mid-slice fractional wall thickening, measured as an index of regional wall motion at the level of the infarct, is shown for sectors 1-6 (Figure 5, right panels). In control MI pigs there was marked reduction in fractional wall thickening across sectors 2–6 (the infarct and border zones). Whilst exosome treated pigs showed some reduction across these sectors after MI, in both sectors centered on the infarct zone there was significant amelioration of the regional wall abnormality (*p* = 0.006 for sector 4 and *p* = 0.013 for sector 5, both by ANOVA). Again, for both sectors the amelioration of regional wall abnormality by exosomes was similar at both day 7 and day 28 post-MI. There was no difference between control and exosome treated pigs for fractional wall thickening of sectors outside the infarct zone (sectors 1-3 and 6 shown in Figure 5) or across sectors 7-12 (data not shown).

**Figure 6:**
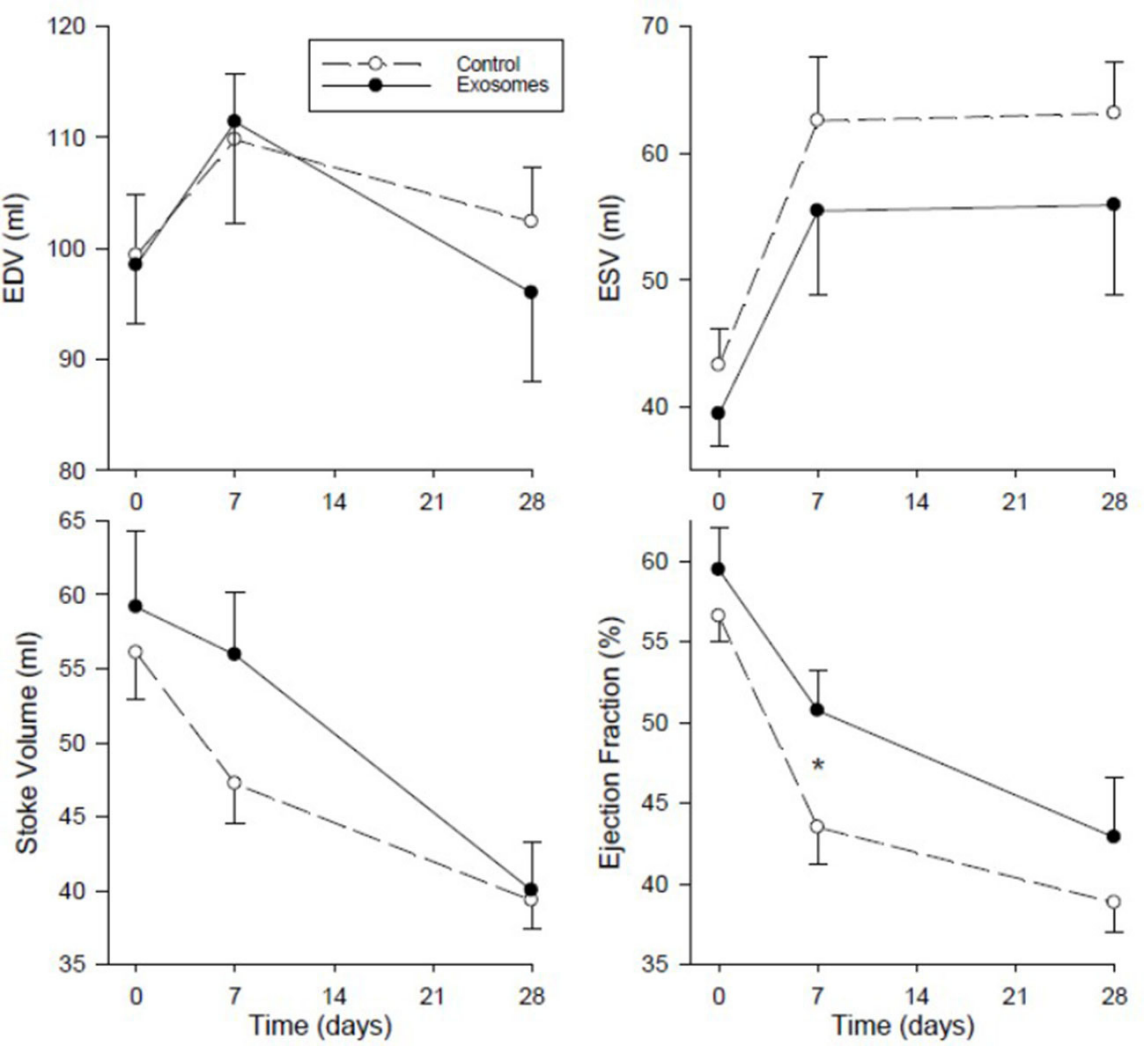
Serial left ventricular end diastolic volume (EDV), end systolic volume (ESV), stoke volume, and ejection fraction measured by MRI in ten pigs receiving 7 days intravenous administration of exosomes (•) following MI and ten pigs vehicle control (°) treated pigs. Values shown are mean ± SEM. Significant differences at time-matched points between the exosomes and control pigs are indicated as follows: *p < 0.05.

Global LV function assessed as LV volumes and ejection fraction by MRI are shown in Figure 6. End diastolic volume (EDV) showed no clear pattern of response across MI in either group and with no significant difference between control and exosome treated pigs. End systolic volumes (ESV) showed an increase of ~50% from baseline following MI in the control MI group with a trend for this increase to be ameliorated in the exosome treated pigs but this difference did not attain statistical significance. There was a fall in both stroke volume and LVEF in response to MI in all pigs. For LVEF, the fall was significantly less with exosome treatment compared with control (*p* = 0.008 by ANOVA) with significant amelioration present by day 7 post-MI (*p* < 0.05). Stroke volume did not differ significantly between control and exosome treated pigs (Figure 6).

**Figure 7:**
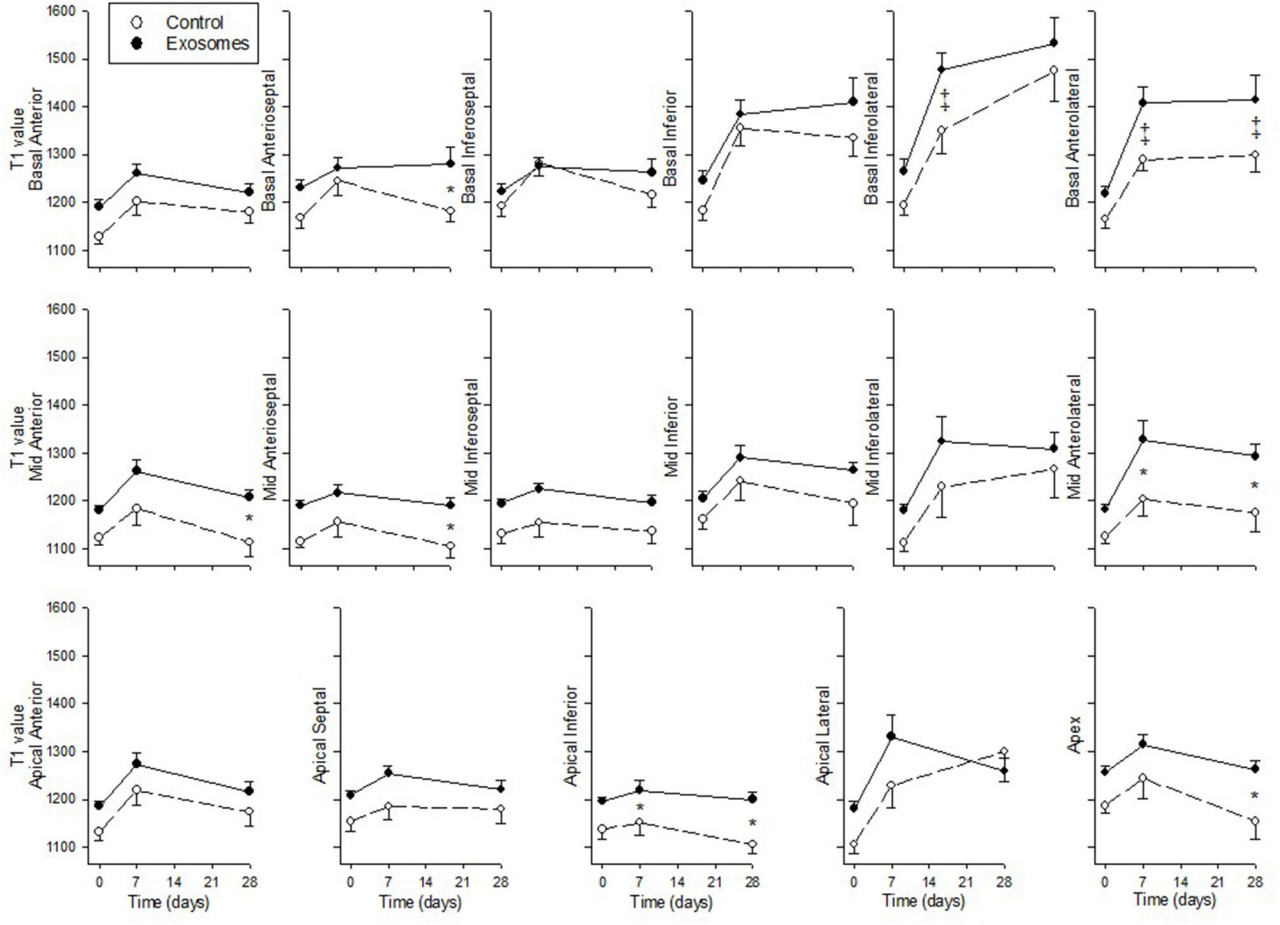
Serial cardiac MRI T1 MOLLI values across 17 segments in ten pigs receiving 7 days intravenous administration of exosomes (•) following MI and ten pigs vehicle control (°) treated pigs. Values shown are mean ± SEM. Significant differences at time-matched points between the exosomes and control pigs are indicated as follows: *p < 0.05 and ‡*p* < 0.001.

Native T1 values derived from MOLLI, an index of cardiac fibrosis, are shown across 17 segments in Figure 7. All three segments specific for LCX occlusion (21) showed a significant increase in T1 values in response to exosome treatment in basal inferolateral (*p* = 0.003 by ANOVA), basal anterolateral (*p* = 0.002) and mid anterolateral (*p* = 0.019) segments. There was also significantly greater increase in T1 values in exosome treated pigs compared with control in one border zone (mid anterior, *p* = 0.01 by ANOVA) and in four remote zones (basal anteroseptal, *p* = 0.03; mid anteroseptal, *p* = 0.01; apical inferior, *p* = 0.008; and apex, *p* = 0.013). The remaining nine zones showed no significant difference between exosome and control treated pigs.

## Discussion

The observations that MSCs exert cardiac protection and repair via their secretome and that the active component(s) of MSC conditioned medium is/are carried by exosomes underpinned our test of the efficacy of MSC exosomes in a large animal model of MI when administered systemically by the convenient method of IV bolus injection. Results from this study using the widely accepted clinically relevant LCX pig model of MI show that 7 days of IV exosomes results in clear reduction (30–40%) of infarct size measured at both 7 and 28 days post-MI, despite near identical release of hs Troponin T over the first 7 days. Together with reduced infarct size, exosome treatment reduced transmurality and lessened wall thinning in the infarct zone. Exosome treated pigs showed relative preservation of LV function with significant amelioration of falls in fractional wall thickening compared with control. However, global measures of LV function were less protected by exosome treatment with amelioration of LVEF only apparent at Day 7 whilst a trend for amelioration of increased LVESV did not reach statistical significance. It is possible that greater preservation of global LV function may have been attenuated by increased cardiac fibrosis as T1 values showed significant increase in the exosome pigs compared to control in the infarct related segments along with increases observed in some but not all border zone and remote segments. Whereas, prolonged aggressive fibrotic responses are clearly deleterious to LV function, the relatively localized modest increase in fibrotic response observed in the current experiment may have helped limit infarct and border zone thinning and expansion. Taken together, these results show clear effects of IV exosomes administered over the first 7 days post-MI to reduce infarct size with relatively preserved cardiac function compared to control treated infarct pigs.

We have previously used the pig LCX model of MI to demonstrate that MSC conditioned medium reduced infarct size and improved cardiac function under a number of experimental settings (8, 9). Acute studies (only examining hearts 5.25 h post-I/R) examining a combination of IV and intra-coronary administration of conditioned medium resulted in both decreased infarct size and improved function following LCX I/R (8). A follow-up study examined effects of conditioned medium administered IV for 7 days following LCX permanent ligation with pigs followed for three weeks post-MI with reduced infarct size and preserved systolic and diastolic function (9). Fractionation studies of the conditioned medium revealed that only products >1000 kDa provided cardioprotection in a mouse model of cardiac I/R injury (8). We then demonstrated that MSCs secreted 50–200 nm particles characterized as exosomes and that purified exosomes administered IV reduced infarct size measured 24 h post-MI in a mouse model of I/R injury (10, 11) and improved cardiac function measured up to 28 days post-MI (11). This led us to conclude that MSC mediated its cardioprotective paracrine effects by secreting exosomes. Others have also demonstrated efficacy of exosomes in rodent models. Cardiac progenitor cells (CPCs) depleted of exosomes did not reduce infarct size in a mouse model of MI induced by ligation of the left anterior descending (LAD) coronary artery whereas when intact exosomes were administered into the border zone myocardium this significantly reduced infarct size measured 48 h post-MI (22). In another species, MSC exosomes injected intra-myocardially in the infarct border zone enhanced blood flow recovery, reduced infarct size and improved cardiac systolic and diastolic performance in a rat LAD coronary artery ligation model of MI (23). The reduction in infarct size was apparent 28 days post-MI but not in rats euthanized 2 days post-MI (23). Given these promising results in rodents, studies of exosome efficacy in large animal models are required before translation to clinical studies. While other investigators have shown exosomes secreted by cardiosphere-derived cells delivered directly to the myocardium can reduce infarct size (24)—discussed in more detail below—our current results are the first to demonstrate that MSC exosomes administered via the more practical and less invasive IV route are efficacious in reducing infarct size with improvement in regional and global LV function.

Choice of methodology for modeling MI is critical if you are wanting to demonstrate effects on LV functional parameters such as LVEF. Although many porcine models can and do result in clear infarcts and some degree of LV dysfunction, the dysfunction is often mild to moderate due to the need to balance the degree of ischemic damage and resultant infarct size against the propensity for animals to suffer lethal arrhythmia. Ligation of the LAD after the 2nd diagonal to create infarct sizes of ~13% has been reported in some studies to have no effect on LVEF measured at 4 weeks (25, 26). Others have reported clear differences between ligating the LAD and LCX with LAD infarcts of ~15% inducing only modest reduction of LVEF (53.4% at 96 h compared with 65.5% at baseline) but proximal LCX infarcts of 17.9% reducing LVEF to 36.8% (27). Another study with even smaller LCX infarcts (10.6%) resulted in a fall in LVEF to 54.1 ± 4.6% at 3 months, a significant reduction from ~70% measured at baseline and in sham pigs (28). Methodology employed in this study is identical to that previously reported from our own Institute (29) and the Utrecht de Kleijn lab (co-author) (9, 30) whereby proximal LCX ligation in similar sized pigs undergoing identical anesthetic protocols consistently induced infarcts in the range of 13–17% and consistently resulted in reduction of LVEF to levels in the range of 28–40% depending on when LVEF was measured (9, 29, 30). In agreement with these previous studies, the vehicle treated group in the current study LVEF was 56.5 ± 4.9 at baseline and was reduced to 43.5 ± 7.2% when measured at 7 days and further reduced to 38.8 ± 5.64 by day 28. Thus, although our infarct sizes were relatively modest (12.5 ± 4.3%) there was a significant reduction in measured LVEF and also an ability to show significant amelioration of the decline in LVEF by exosome treatment.

In order to be practical for treating patients, either immediately after MI or in a serial manner for days after MI, then the route of administration of exosomes is critical. A recent review (31) highlights the wide variety of different administration routes that have been used to evaluate cell based, secretome and vesicle/exosomes treatment in the post-MI setting. These range from highly invasive methods including trans-epicardial and myocardial patches, which invariably require an open chest procedure, to intracoronary, which is convenient at the time of percutaneous intervention but impractical for administration of repeat doses. Novel devices such as the NOGA mapping and injection system allow trans-endocardial administration of intra-myocardial injection and these have been trialed for administering either cell-based therapy (32) or cardiosphere-derived exosomes (24) in pig models. However, this delivery route is not risk free and impractical if repeat treatment/injections are required. Of note in the exosome study, the exosomes were administered by two different routes, namely intra-coronary and intramyocardially (via the NOGA system) and whilst the direct injection into the border zone myocardium did demonstrate beneficial effect, the intra-coronary route had no significant beneficial effects (24). Clearly, in order to allow repeat administration and be more cost effective with less complication rates associated with treatment, a less invasive route of administration such as IV bolus is preferable. Of note, our earlier mouse model work showed infarct reduction following a single IV injection of exosomes (10, 11). Furthermore, both our previous pig study with conditioned medium (9) and the current study administering exosomes both used repeated twice daily IV bolus injections to show effective reduction in infarct size with some improvement on cardiac function.

Timing of administration of exosomes is another key factor. In rats, intra-myocardial injection of extracellular vesicles into the border zone at a single time-point 30 min after coronary ligation had no significant effect on infarct size in a subset of rats euthanized at 2 days post-MI whereas infarct size was significantly reduced when measured 28 days post-MI (23). Thus, a single injection of extracellular vesicles can differentially affect acute vs. chronic infarct size suggesting treatment –induced changes apparent only days or weeks after treatment. Also of interest, Gallet et al. (24) used the NOGA system to inject into the border zone of pigs with well-developed infarcts, namely, a single time-point injection of exosomes administered 28 day post-MI to test whether exosomes could “rescue” a convalescent or established infarct (24). MRI analysis 4 weeks later (8 weeks post-MI) demonstrated exosomes preserved LV volumes and LVEF and decreased scar size and transmurality. Thus, they demonstrated that not only could acute administration of exosomes at 48 h post-MI reduce infarct size but treating convalescent MI 4 weeks after MI partially reversed established scar (24). Of note in the current study, both the peak hsTroponin T and time course over the first seven days was identical between control and exosome treated groups. This suggests that both the initial ischemic assault and degree of early myocardial injury was equivalent between the groups. However, concurrent twice daily treatment with exosomes modified the evolution of the infarct, possibly including tissue necrosis, edema, neutrophil migration, and subsequent inflammatory processes, such that by day 7, the infarct size assessed by MRI was already significantly less in exosome-treated compared with control treated pigs and these differences in infarct size remained though to 28 days post-MI. We had previously shown (9) that pigs treated with IV MSC conditioned medium over the same course as the present study (twice daily injections for 7 days) showed a similar degree of reduction in infarct size measured at 21 days post-MI to the infarct size reduction measured at 28 days post-MI in the present study. However, in our earlier study preservation of LVEF appeared greater being 49.1 ± 3.3% compared with 33.7 ± 4.4% in control (non-CM) treated pigs (9). Although measured at different time-points, LVEF was only modestly improved at day 7 (50.7 ± 2.5% compared with 43.5 ± 2.3%) with less difference apparent by 28 days (42.9 ± 3.7% compared to 38.8 ± 1.8%, not significant) for exosome compared with control pigs, respectively. It may be that, despite large percent reductions in infarct size by exosome treatment, increases in fibrosis, as measured by T1 MOLLI MRI both at the infarct, border zone and remote LV regions in the exosome treated pigs, may have both limited scar size and limited contractile improvement in LV function, at least in this experimental setting.

The present study has not examined specific mechanisms underlying the cardioprotective and regenerative actions of exosomes. However, numerous previous studies have described a plethora of trophic factors by which MSC secretome and exosomes can mediate their actions to reduce tissue injury, protect tissues from further adverse effects and enhance tissue repair (33). We have previously identified, by proteomic analysis of MSC conditioned medium, up to 739 proteins in the secretome including many exosome associated proteins (10). Computational analysis predicts that this secretome has potential to repair injured tissue in the MI setting (34). As lipid vesicles, exosomes represent an ideal vehicle to effect repair and recovery via rapid intracellular delivery of functional proteins, lipids and microRNAs to the target tissue. We have also previously shown that MSC exosomes reduce apoptosis and autophagy in both hypoxic myoblast cells and I/R of isolated rat hearts (35). Others have confirmed protection of cardiomyocytes against hypoxia-reoxygenation injury through autophagy signaling and cell apoptosis (36). A number of *in vivo* rodent studies have demonstrated that exosome treatment post-MI induces significant increase in angiogenesis. In a rat MI model, MSC extracellular vesicles promoted angiogenesis resulting in enhanced blood flow recovery post-MI (23). MSC exosomes increased capillary density and Ki67 positive stained cells and induced cardiomyocyte proliferation resulting in preserved cardiac function, all measured 28 post-MI in a mouse model (37). In our earlier pig studies, MSC conditioned medium treatment resulted in higher myocardial capillary density in the infarct border zones (9). This mechanism of stimulating myocardial angiogenesis in the infarct and/or border zone is also supported by data in a rat model of permanent ligation MI where exosomes derived from CPCs and MSCs both increase blood vessel density in the infarct region (38). In the current study we were not able to confirm blood vessel stimulation as we did not collect tissue samples for histology from all pigs (only a subset of four from each group). Other mechanisms whereby exosomes likely exert their cardiac protection and repair effect are by reducing oxidative stress (11) and modulating the local inflammatory response and environment (11, 39). Taken together, it is likely that multiple mechanisms contribute to exosomes ability to protect, repair, and regenerate myocardium following a prolonged cardiac ischemia and MI.

One potential limitation of this current study is that we did not evaluate any potential immunological response to xenogenic exosomes IV administration. However, it can be noted that these pigs clearly attained net benefit over a month of follow-up. This might imply no immediate cytokine storm was induced which is not surprising given the extensive prior work by our group. Indeed, we have previously administered human MSC exosomes in mice, rats and pigs (11, 40–43). Consistently across all the species, human MSC exosomes did not elicit a pro-inflammatory immunogenic response. Instead, they elicited an anti-inflammatory regenerative immune response that was associated with the therapeutic outcomes. Most relevant to this current study, we have previously reported that MI reduction subsequent to ischemia-reperfusion injury by our MSC exosome preparation was concomitant with a significant decrease in neutrophils and macrophages in the porcine heart tissue (11). This is consistent with anti-inflammatory properties of these MSC exosome (41). In no instances have we needed to use either genetically immune deficient or drug-induced immunosuppression when administering our MSC exosomes.

In conclusion, we demonstrated in pigs undergoing LCX ligation model of MI that MSC-derived exosomes administered by IV route over the first seven days post-MI reduce infarct size and infarct wall thinning despite identical acute hs Troponin T release compared with control treated pigs. There was some evidence of improved regional and global LV function measured by MRI at days 7 and 28 post-MI but significantly increased T1 values across the infarct and non-infarcted myocardium, indicating increased fibrosis in the exosome treated pigs. Early accelerated, yet relatively localized and limited, fibrosis may underlie some the benefits observed. Further studies are required to see if dose, duration and timing of exosome treatment will further improve not only infarct size reduction but improve cardiac function post-MI. This study provides further evidence that MSC exosomes offer promise as a safe, cost effective “off-the-shelf” therapy for cardiac protection and repair following MI.

## Data Availability Statement

The raw data supporting the conclusions of this article will be made available by the authors, without undue reservation.

## Ethics Statement

The animal study was reviewed and approved by National University of Singapore IACUC.

## Author Contributions

CC: principle investigator, hands on experimentation, statistical analysis, lead in drafting and editing manuscript, and submission of manuscript. RRL, TY, and SM: hands on experimentation, critique of manuscript. RCL: production of exosomes, DK: conceptualization, critique of manuscript. SL and AR: conceptualization, funding application, critique of manuscript. All authors contributed to the article and approved the submitted version.

## Conflict of Interest

SL is a Founder Shareholder of Paracrine Therapeutics, a biopharmaceutical company focused on development of stem exosome for regenerative medicine. The remaining authors declare that the research was conducted in the absence of any commercial or financial relationships that could be construed as a potential conflict of interest
